# Regulatory factor identification for *nodal* genes in zebrafish by causal inference

**DOI:** 10.3389/fcell.2022.1047363

**Published:** 2022-10-20

**Authors:** Cencan Xing, Zehua Zeng, Yaqi Li, Bo Gong, Weimin Shen, Roshan Shah, Lu Yan, Hongwu Du, Anming Meng

**Affiliations:** ^1^ Daxing Research Institute, School of Chemistry and Biological Engineering, University of Science and Technology, Beijing, China; ^2^ Laboratory of Molecular Developmental Biology, State Key Laboratory of Membrane Biology, Tsinghua-Peking Center for Life Sciences, School of Life Sciences, Tsinghua University, Beijing, China; ^3^ Guangzhou National Laboratory, Guangzhou, China

**Keywords:** *nodal*, Eomes, β-catenin, Smad2, zebrafish

## Abstract

Activation of *nodal* genes is critical for mesoderm and endoderm induction. Our previous study reported that zebrafish *nodal* genes *ndr1*/*squint* and *ndr2*/*cyclops* are coordinately regulated by maternal Eomesa, Hwa-activated β-catenin (Hwa/β-catenin) signaling, and Nodal autoregulation (Nodal/Smad2) signaling. However, the exact contribution and underlying mechanisms are still elusive. Here, we applied “causal inference” to evaluate the causal between the independent and dependent variables, and we found that Hwa/β-catenin and Smad2 are the cause of *ndr1* activation, while Eomesa is the cause of *ndr2* activation. Mechanistically, the different *cis*-regulatory regions of *ndr1* and *ndr2* bound by Eomesa, β-catenin, and Smad2 were screened out *via* ChIP-qPCR and verified by the transgene constructs. The marginal GFP expression driven by *ndr1 transgenesis* could be diminished without both maternal Eomesa and Hwa/β-catenin, while Eomesa, not β-catenin, could bind and activate *ndr2* demonstrated by *ndr2* transgenesis. Thus, the distinct regulation of *ndr1*/*ndr2* relies on different *cis*-regulatory regions.

## Introduction

The Nodal proteins, which belong to a member of the transforming growth factor β (TGFβ) family, play an essential and conserved role in mesoderm and endoderm induction and specification (Schier and Talbot, 2005; Tian and Meng, 2006; Schier, 2009; Zinski et al., 2018). In the mouse, the zygotic homozygous mutants of the only *Nodal* gene failed to form most mesendodermal tissues (Zhou et al., 1993; Conlon et al., 1994). The disruption of Nodal-related genes in Xenopus led to severe defects in mesendoderm induction (Jones et al., 1995; Joseph and Melton, 1997; Osada and Wright, 1999; Agius et al., 2000; Takahashi et al., 2000; Luxardi et al., 2010). In the zebrafish, the removal of two *nodal*-related ligands, namely, *ndr1/squint (sqt)* and *ndr2/cyclops(cyc)*, leads to the loss of endodermal tissues and most mesodermal tissues (Feldman et al., 1998).

As diffusible proteins, Nodal could propagate its expression in the adjacent marginal cells by Nodal autoregulation (Jones et al., 1996; Chen and Schier, 2002; Schier, 2009; Muller et al., 2012; Muller et al., 2013). Once Nodal signaling is activated, the intracellular effectors Smad2 and Smad3 could form a complex with Smad4 and translocate into the nucleus with the help of FoxH1 or/and other transcription factors and activate the target genes, including *nodal* genes themselves, which harbor the Nodal-responsive elements (NRE) within the first intron (Adachi et al., 1999; Norris and Robertson, 1999; Osada et al., 2000; Fan et al., 2007; Liu et al., 2011). However, the initiation of zygotic *nodal* genes is elaborately regulated in vertebrate embryos.

In both zebrafish and Xenopus, zygotic *nodal* transcripts are triggered by maternally controlled dorsally localized nuclear β-catenin (Feldman et al., 1998; Kofron et al., 1999; Kelly et al., 2000; Tao et al., 2005; Bellipanni et al., 2006). In maternal *huluwa* (*hwa*), mutant embryos, which lose the nuclear β-catenin in the dorsal blastomere (Yan et al., 2018), are also unable to initiate *ndr1* in the dorsal blastodermal margin (Xing et al., 2022), providing an essential role of maternal Hwa-activated β-catenin signaling in activating zygotic *nodal* in dorsal blastomere. In Xenopus, the maternal expressed T-box transcription factor VegT collaboratively functions with β-catenin to trigger zygotic *nodal* genes in the vegetal cell mass (Zhang et al., 1998; Kofron et al., 1999; Agius et al., 2000; Takahashi et al., 2000; Rex et al., 2002; Xanthos et al., 2002). In the zebrafish, the maternal T-box transcription factor Eomesodermin (Eomesa) was assumed to be a zebrafish functional counterpart of frog VegT, as Eomesa could directly activate *ndr1* and *ndr2* in ventral and lateral blastodermal margins (Xu et al., 2014), which was further verified as sole maternal factors as well as maternal Hwa/β-catenin signaling for zygotic *nodal* genes expression in zebrafish embryos (Xing et al., 2022), in which the maternal Eomesa, maternal Hwa-activated β-catenin signaling, and Nodal autoregulation are required to weave the spatiotemporal and dynamical expression of *ndr1* and *ndr2* in zebrafish. However, it is unclear how these factors differentially contribute to activating the *nodal* genes with preference.

In this study, we constructed causal graphical models to uncover the cause of *ndr1* and *ndr2* activation and screened out the genomic sequences of *ndr1* and *ndr2* bound by maternal Eomesa, maternal Hwa-mediated β-catenin, and Nodal autoregulation-mediated Smad2, and further verified the *cis*-regulatory regions by transgenic constructs, providing pieces of evidence to precisely understand the dynamical activation of *ndr1* and *ndr2* during the mesendoderm induction in early zebrafish embryos.

## Materials and methods

### Zebrafish strains and embryo incubation

The zebrafish Tuebingen strain was used as WT fish and for generating mutants. M*eomesa*, M*hwa*, and M*eomesa*;M*hwa* mutant embryos were genotyped and obtained as previously described (Xing et al., 2022). Embryos were maintained in Holtfreter’s water at 28.5°C. Developmental stages of the maternal mutant embryos were indirectly determined by observation of WT embryos (Kimmel et al., 1995), which were born at the same time and incubated under identical conditions. For the Nodal signaling inhibitor SB431542 (SB) treatment, one-cell stage embryos (10 min postfertilization) were incubated in Holfreter’s water with 1% DMSO (control) or 50 μM SB and harvested or observed at desired stages. All experiments were approved by Tsinghua University Animal Care and Use Committee.

### Double fluorescence *in situ* hybridizations, imaging, and quantification

Whole-mount high-resolution double fluorescence *in situ* hybridization (DFISH) was carried out using the protocol provided by J. Gage Crump lab, as described by Welten et al. (2006) and Zuniga et al. (2010), with two modifications: antibody concentrations were 1:500 anti-fluorescein-POD and 1:1000 anti-digoxigenin-POD (without preadsorbing the antibody with prehybridized zebrafish embryos), and incubation time with fluorescein tyramide was 3 h in the dark. The DIG-labeled *ndr1* probe and fluorescein-labeled *ndr2* were used for DFISH.

Embryos were embedded in 1% low-melting-point agarose and imaged *via* Zeiss light-sheet Z.1 microscopy using a W Plan-Apochromat 20× objective at 0.5× zoom. Embryos were positioned with lateral views, and two lateral images were acquired for each embryo: one at the brightest view, and the other, rotated 180°. Each lateral view image generated by online dual side fusion included ∼250 z-slices with 1 μm interval. Then, two lateral views were fused by Multiview Process using ZEN (2014 SP1, black edition). The final image file (a size of ∼3 GB) per embryo was manually rotated in Imaris X64 9.0 to acquire the maximum intensity projections of animal views with dorsal to the right (indicated by the enhanced signal in the dorsal margin of WT and M*eomesa* embryos), which were captured after reset of all channels for further measurements.

For quantification of FISH, the maximum intensity projections were opened in Fiji to manually draw a circular polygonal region of interest (ROI) covering the entire embryo margin to measure the raw intensity profile (in uncalibrated optical density values, OD) per embryo. The staining dots in the marginal region were dispersedly distributed with blank, which could be a favorable control for the background subtraction. So, the minimal intensity (the intensity of the blank) in the marginal region (in a smaller and rigorous ROI) of the embryo was removed from the total intensity. The number of embryos assessed in FISH with final total intensity of *ndr1* or *ndr2* is presented in Supplementary Table S7.

### Linear regression model

We assume the expression levels of *ndr1* and *ndr2* follow the linear regression model with maternal Eomesa, Hwa/β-catenin, Nodal/Smad2, and time:

$$Y_{ndr1}=\alpha_{1}X_{eomesa}+\alpha_{2}X_{\beta -Catenin}+\alpha_{3}X_{Smad2}+\alpha_{4}X_{Time}+\alpha_{0},$$

$$Y_{ndr2}=\beta_{1}X_{eomesa}+\beta_{2}X_{\beta -Catenin}+\beta_{3}X_{Smad2}+\beta_{4}X_{Time}+\beta_{0}.$$

To estimate the value of the coefficients α and β and the t-test of the coefficient, we performed a maximum likelihood estimation using the statsmodel library in Python (Seabold and Perktold, 2010).

For the analysis of the regression coefficients of Hwa/β-catenin and Nodal/Smad2 in *ndr1*, we assumed that the following regression relationship was satisfied:

$$Y_{ndr1}\left(i\right)=\alpha_{1}X_{eomesa}\left(i\right)+\alpha_{2}X_{\beta -Catenin}\left(i\right)+\alpha_{3}X_{Smad2}\left(i\right)+\alpha_{0},$$

where i denotes the different time points.

Similarly, we assumed that the following regression relationship of maternal Eomesa and Nodal/Smad2 in *ndr2* was satisfied:

$$Y_{ndr2}\left(i\right)=\beta_{1}X_{eomesa}\left(i\right)+\beta_{2}X_{\beta -Catenin}\left(i\right)+\beta_{3}X_{Smad2}\left(i\right)+\beta_{0},$$

where i denotes the different time points.

### Causal inference

It is assumed that we want to find the causal effect of the existence of maternal Eomesa, Hwa/β-catenin, Nodal/Smad2, and the value of time on the outcome of *ndr1* and *ndr2*. For *ndr1*, to define the causal effect, we consider two situations: Situation 1 (real situation): Where the existence of Hwa/β-catenin, Nodal/Smad2, and the value of time are changed, and the expression of *ndr1* is observed. Situation 2 (counterfactual situation): Where the existence of Hwa/β-catenin, Nodal/Smad2, and the value of time are unchanged (but the expression of *ndr2* and the existence of Eomesa are consistent with real situation). The causal effect is the difference between the expression of *ndr1* attained in the real situation and the counterfactual situation:

$$E\left[Y_{\left(ndr1\right),real,X_{\beta -Catenin}=1{,X}_{Smad2}=1}\right]-E\left[Y_{\left(ndr1\right),real,X_{\beta -Catenin}=0{,X}_{Smad2}=0}\right].$$

In other words, X causes Y if changing X leads to a change in Y, keeping everything else constant. Changing X while keeping everything else constant is called an intervention, and it is represented by a special notation, do(X) .

Formally, causal effect is the magnitude by which Y is changed by a unit interventional change in X:

$$E\left[Y_{\left(ndr1\right)}|do\left(X=1\right)\right]-E\left[Y_{\left(ndr1\right)}|do\left(X=0\right)\right].$$

To estimate the target quantity of the given observed variable, a backdoor criterion was introduced. If all common causes of the existing X and the outcome Y are observed, then the backdoor criterion implies that the causal effect can be identified by conditioning on all the common causes:

$$E\left[Y_{\left(ndr1\right)}|do\left(X=1\right)\right]=E_{w}E\left[[Y_{\left(ndr1\right)}|X=1,\;W=w\right],$$

where W refers to the set of common causes (the expression of *ndr2* and the existence of Eomesa). After the identification of the correct estimand for the target quantity based on the causal model and estimation of the target estimand, we performed a refutation test by adding a random common cause variable (the estimation method does not change its estimate after we add an independent random variable as a common cause to the dataset), replacing treatment with a random (placebo) variable (the estimated causal effect should go to zero when we replace the true treatment variable with an independent random variable), and removing a random subset of the data (the estimation method does not change its estimate after we add an independent random variable as a common cause to the dataset) using the DoWhy library in Python (Sharma and Kiciman, 2020).

### Chromatin immunoprecipitation quantitative real-time PCR

ChIP assay was performed as previously described (Listerman et al., 2006) with minor modifications: 1) the embryos (1,000 embryos per sample) were cross-linked with 1.85% formaldehyde (Amresco, 0493) for 15 min; 2) Sepharose beads were replaced by magnetic beads (Sigma, 16–663X) for convenience. The antibodies were mouse anti-Myc (Santa Cruz, sc-40, 10 μg), mouse IgG (Biyuntian, A7028, 10 μg, as control), rabbit anti-β-catenin (Cell Signaling Technology, #8408, 20 μl), rabbit anti-Smad2/3 (Cell Signaling Technology, #3102, 30 μl) (Liu et al., 2011), and rabbit IgG (Biyuntian, A7016, 0.125 μg, as control). Following ChIP, the purified DNA was used for qRT-PCR using specific primers (Supplementary Table S4, S5) with some changes according to Hong et al, (2011). Percentage inputs (% input) was calculated by “% Input = 2^(-ΔCt [normalized ChIP]),” where ΔCt [normalized ChIP] was acquired from “(Ct [ChIP] - (Ct [Input] -Log2 (input dilution factor))” with the input dilution factor of 10 in the ChIP. Standard deviation (S.D.) and multiple *t*-tests were analyzed using GraphPad Prism 7 from two independent experiments.

### Constructs and microinjection

For making transgene constructs, the putative regulatory regions of *ndr1* and *ndr2* were amplified using specific primers (Supplementary Table S6), and the resulting fragments were ligated to drive GFP expression (Figures 3, 4). The transgene constructs were purified and injected into embryos at the one-cell stage. GFP expressions in embryos at 4.3–5 hpf were observed and photographed under Olympus MVX10 fluorescence microscopy.

## Statistics

The graphs and *t*-tests were finished with GraphPad Prism 7. Error bars were represented as mean ± S.D. *p* values are two-sided. Significance levels were indicated by non-significant (ns), *p* > 0.05; *, *p* < 0.05; **, *p* < 0.01; and ***, *p* < 0.001.

## Results

### The causal inference model reveals the regulatory factors of *ndr1* and *ndr2*

**FIGURE 1:**
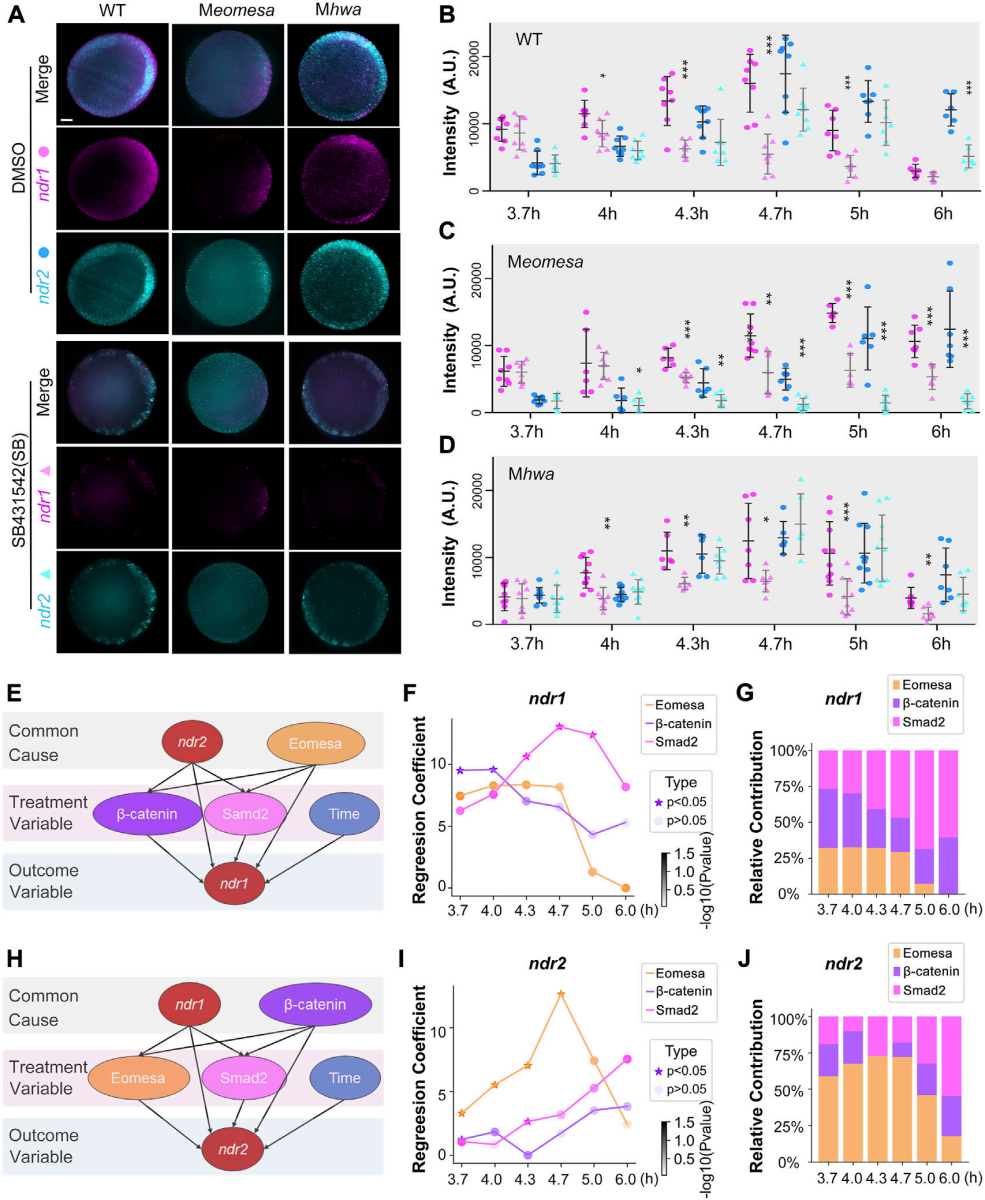
Regulatory factors of *ndr1* and *ndr2* analyzed by a causal graphical model. **(A–D)** Dynamic fluorescence intensities of *ndr1* and *ndr2* quantified for model analysis. **(A)** Representative expression pattern of *ndr1* and *ndr2* at 4.7 hpf detected by double fluorescence *in situ* hybridization (FISH). Embryos were treated with 1% DMSO or 50 μM SB431542 (SB) and fixed at indicated stages. All embryos are shown in animal-pole view with dorsal to the right; the dorsal is indicated by the expression pattern of *ndr1*/*ndr2*. It is of note that under SB treatment, the *ndr1*, not *ndr2*, could be detected in M*eomesa* embryos, while both *ndr1* and *ndr2* were present in M*hwa* embryos. Scale bars, 100 μm. **(B–D)** Quantification of fluorescence intensities of *ndr1* and *ndr2* at indicated stages. In total, 7–10 embryos for each group were measured by Fiji for the total intensities. Pink dots, *ndr1* intensities; blue dots, *ndr2* intensities (circles for the DMSO group, and triangles for the SB group). It is of note that in M*eomesa* treated with DMSO, *ndr2* intensities rise following the intensity increase of *ndr1*. Error bars represent s.d. for all embryos in each group. The asterisks show a significant difference between the SB group and the DMSO group. *p < 0.05; **p < 0.01; ***p < 0.001; not shown, not significant. **(E–G)** Hwa/β-catenin and Nodal/Smad2 could be the treatment variable factors for *ndr1* expression during the time series. **(E)** Causal graphical model for *ndr1* expression level. The causal inference was used to evaluate the causal between independent and dependent variables based on the linear regression model. The *p*-value of the regression coefficient of the independent variable (Eomesa) > 0.2 has no significant effect on the dependent variable *ndr1*, while the *p*-value of the regression coefficient of the independent variable (Hwa/β-catenin, Nodal/Smad2) < 0.2, indicating that these factors have a high contribution in the expression of *ndr1*. **(F)** The regression coefficient of Eomesa, β-catenin, and Smad2 on *ndr1* at different time points. The *p*-value<0.5 was marked as a star. All coefficients were scaled down by a factor of 1000 for visualization. **(G)** The relative contribution of factors to *ndr1* at different time points. **(H–J)** Eomesa and Smad2 could be the treatment variable factors for *ndr2* expression during the time series. The data presentation was similar to those described in **(E–G)**.

Based on our previous study, the expressions of *ndr1* and *ndr2* are hypothesized to be mainly regulated by three factors, that is, maternal *eomesa*, maternal *hwa*-activated β-catenin signaling, and Nodal autoregulation (Xing et al., 2022). To quantitatively explore the dynamic contribution of these three factors to the expression of *ndr1* and *ndr2* during several time points, we first performed intensity compute of *ndr1* and *ndr2* in the maternal *eomesa* (M*eomesa*), maternal *hwa* (M*hwa*), and wildtype (WT) embryos, as well as in the control group with the inhibition of Nodal autoregulation (SB431542 treatment). The expression levels of *ndr1* and *ndr2* were detected by the DFISH, imaged using light-sheet fluorescence microscopy (LSFM), and measured by the software Fuji (Figures 1A and B and Supplementary Table S7), which show a similar expression pattern to the previous reports (Meno et al., 1999; Xing et al., 2022) and simultaneously provide the quantified data for further modeling.

We applied “linear regression” and “causal inference” to evaluate the causal between the independent and dependent variables (Pearl, 2010). To reveal the relationship between variables, we constructed a multiple linear regression model with *ndr1* and *ndr2* intensities as dependent variables, respectively, and Eomesa, Hwa/β-catenin, Smad2, and time as independent variables. For the linear regression model of *ndr1* (Supplementary Table S1), the *p*-value of the regression coefficient of the independent variable Eomesa >0.2 has no significant effect on the dependent variable *ndr1*, indicating that Eomesa may be the common cause for the outcome variable *ndr1*. For *ndr2* (Supplementary Table S2), the *p*-value of the regression coefficient of the independent variable Hwa/β-catenin > 0.2 has no significant effect on the dependent variable *ndr2*, indicating that Hwa/β-catenin may be the common cause for the outcome variable *ndr2*.

Then, a causal graphical model was constructed for *ndr1* with Hwa/β-catenin, Smad2, and time as treatment variables and *ndr2* and Eomesa as the common causes (Figure 1C). The causal inference was performed using the backdoor criterion, and estimators were constructed using the linear model. Refutation tests by adding a random common cause variable, replacing treatment with a random (placebo) variable, and removing a random subset of the data were used to check its robustness to assumptions (Supplementary Table S3). It is of note that the “placebo_treatment_refuter = 0” indicates the model passes the refutation test and works. Similarly, the *ndr2* causal graphical model was constructed with Eomesa, Smad2, and time as treatment variables and *ndr1* and Hwa/β-catenin as the common causes (Figure 1H). Both causal graphical models pass the refutation test (Supplementary Table S3), which not only proves the correlation between *ndr1*, Hwa/β-catenin, and Nodal/Smad2 and the correlation between *ndr2*, Eomesa, and Nodal/Smad2, but also suggests that Hwa/β-catenin and Nodal/Smad2 can be the cause of *ndr1* expression levels, while Eomesa and Nodal/Smad2 can be the cause of *ndr2* expression levels.

We further calculated the variation of regression coefficients of Hwa/β-catenin and Nodal/Smad2 on *ndr1* at different time points, and we found that the regression coefficients and the percentage contribution of Hwa/β-catenin gradually decreased during development (Figures 1F and G); meanwhile, those of Nodal/Smad2 gradually increased, indicating that the control of *ndr1* expression was alternately transitional from Hwa/β-catenin to Nodal/Smad2. For *ndr2*, the coefficient variations of Eomesa continuously and steadily increased from 3.7 hpf to 4.7 hpf (Figures 1I and J), but the regression coefficients and the percentage contribution of Eomesa gradually decreased at later stages and were accompanied by the gentle rise of Nodal/Smad2.

### Distinct locations of regulator regions at *ndr1* and *ndr2* loci

**FIGURE 2:**
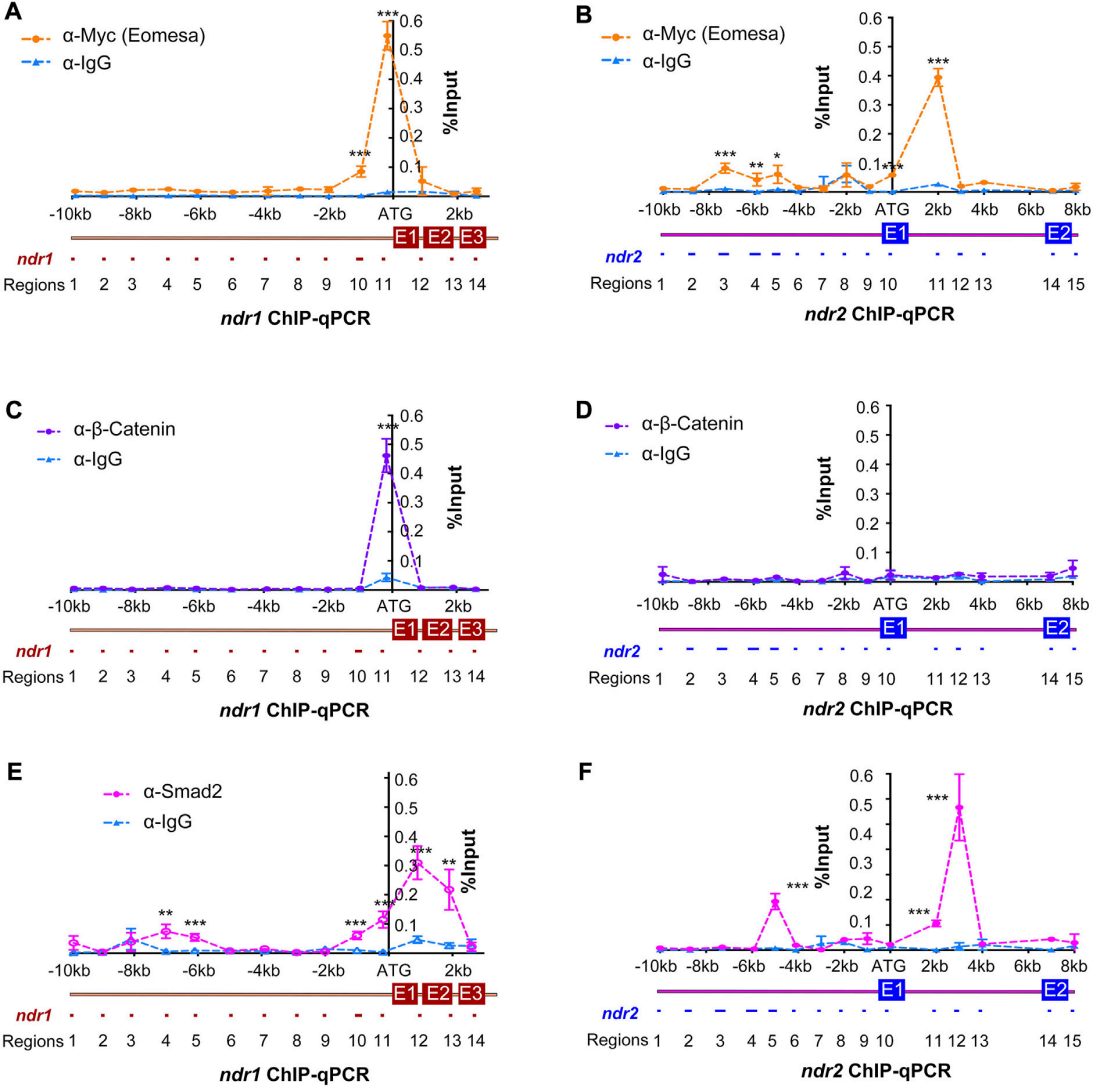
Identification of binding regions in *ndr1* and *ndr2* loci for Eomesa, β-catenin, and Smad2. Uninjected WT embryos or those injected with 50 pg *Myc-eomesa* mRNA were harvested at 4.3–5 hpf for chromatin immunoprecipitation using anti-Myc **(A and B)**, anti-β-catenin **(C and D)**, anti-Smad2 **(E and F)**, or IgG antibody (Ab). The immunoprecipitated chromatin was used for quantitative PCR analysis using specific primers targeting different regions, as illustrated. The PCR results were normalized with the input genomic DNA with dilution factor (e.g., 1:10). The x-axis showed the genomic organization of *ndr1* or *ndr2* with amplified regions numbered. The translation start site was designated as position +1. The y-axis indicated the average percentage (±SEM) of amplified product relative to input DNA (% input) based on two independent experiments. Statistical significance levels: *, *p* < 0.05; **, *p* < 0.01; ***, *p* < 0.001; ns, nonsignificant.

To better understand how *ndr1* and *ndr2* are mechanistically regulated by Eomesa, β-catenin/Lef, and Smad2, the binding activity and regions were screened out by chromatin immunoprecipitation–quantitative PCR (ChIP-qPCR) analysis in zebrafish embryos at 4.3–5 hpf. Using immunoprecipitated DNA with a size range of 0.3–1 kb as the template, 14 *ndr1* regions (ndr1R1–14) and 15 *ndr2* regions (ndr2R1–15) (Figure 2), ranging from 60 to 250 bp, were amplified with specific primer pairs (Supplementary Table S4, S5). Results revealed that the promoter region (ndr1R11) of *ndr1* was significantly bound by Eomesa (tagged with Myc), β-catenin, and Smad2, while Eomesa also occupied the upstream region ndr1R10 and Smad2 occupied the upstream regions ndr1R4, ndr1R5, ndr1R10 and the gene body regions ndr1R12 and ndr1R13 at the *ndr1* locus. Concerning the *ndr2* locus, the promoter region ndr2R10 was significantly bound only by Eomesa, and several upstream regions and gene body regions appear occupied by Eomesa and Smad2. However, β-catenin lacked binding peaks in the tested regions of *ndr2*, which is consistent with the observation that the *ndr2* expression level was not significantly affected in *Mhwa* mutants (Figures 1A and B). It is of note that the *ndr1* and *ndr2* genomic DNA reported to be bound by Eomesa (Xu et al., 2014) are included in ndr1R11 and ndr2R13 (Figures 2A and B), and the ndr1R12 region covers the first intron of *ndr1* predicted to be bound by Smad2 (Fan et al., 2007).

### Verification of regulatory regions of *ndr1 via* transgenesis

**FIGURE 3:**
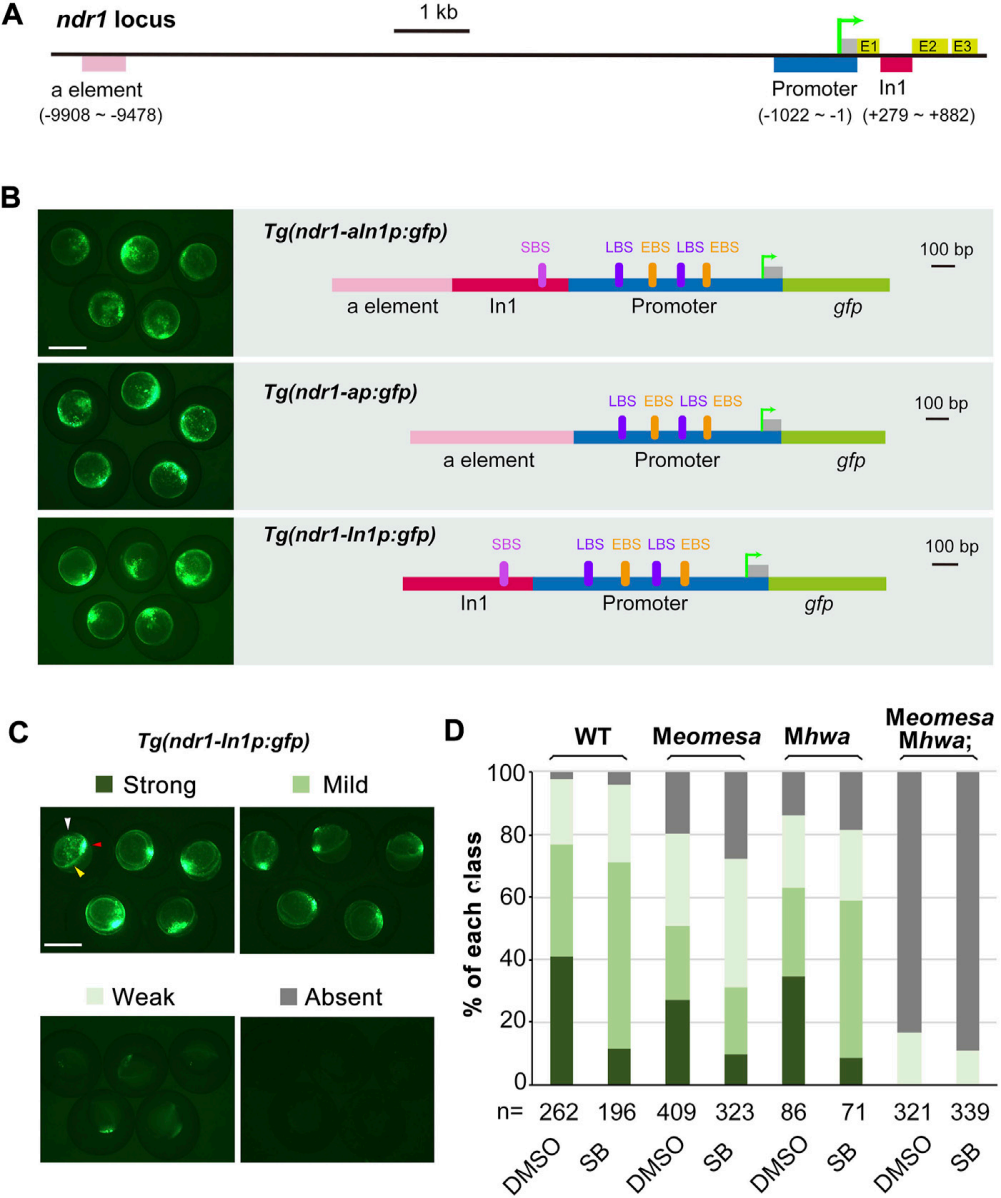
Regulation of *ndr1* expression through transient transgenic assay. **(A and B)** Genomic organization of *ndr1* locus **(A)** and composition of transgenes **(B)**. The translation start site was designated as position +1. E, exons; In1, intron 1. EBS, Eomes-binding site; LBS, Lef/β-catenin binding site; SBS, Smad2-binding site. **(C and D)** Categorization of *ndr1* transgenes in zebrafish embryos. *Tg(ndr1-In1p:gfp)* DNA was injected into one-cell stage WT or mutant embryos (50 pg/embryo), and GFP was observed at 4.3–5 hpf. For SB treatment, injected embryos were incubated in 50 μM SB or 1% DMSO (control) until observation. Based on GFP intensity in the blastodermal margin, embryos were categorized into four classes, as shown for WT embryos (top panel). The expression domain in the blastodermal margin is indicated by yellow heads, and the domain in the dorsal margin is indicated by red arrowheads in typical embryos. The presumably nonspecific signals are indicated by white arrowheads. Scale bars: 500 μm. The bar graphs (bottom) show the ratios of embryos in each class in WT or different mutants. n, the number of observed embryos.

To test the sufficiency of the identified major regions screened out by ChIP-qPCR (Figure 3) for initiating and maintaining *ndr1* expression, several genomic fragments amplified from each gene with specific primers (Supplementary Table S6) were used to construct GFP reporter transgenes (Figures 3A and B). The transgene constructs were individually injected into one-cell stage WT embryos, and GFP expression was observed at 4.3–5 hpf. For the *ndr1* locus, the distal enhancer “a element” reported before (Fan et al., 2007), the 1,022-bp proximal promoter with 5′UTR (p), which embodies ndr1R11 with two putative Eomes-binding sites (EBS) and two putative Lef-binding sites (LBS), plus the 604-bp *ndr1* intron 1 fragment (In1), which contains ndr1R12 with a putative Smad-binding site (SBS), were selected and fused to the *gfp* coding sequence to make the *Tg(ndr1-aIn1p:gfp)*, *Tg(ndr1-ap:gfp)*, and *Tg(ndr1-In1p:gfp)* construct (Figure 3B). Although *Tg(ndr1-In1p:gfp)* did not include the previously identified distal enhancer “a element” of *ndr1* (Fan et al., 2007), it allowed GFP to express in the blastodermal margin with an expanded domain in the dorsal margin in over 70% of WT embryos (Figures 3B and C), the pattern of which mimicked endogenous *ndr1* expression. The ratio of embryos with strong GFP expression in correct domains was reduced in M*eomesa* or M*hwa* mutant embryos, while no embryos showed strong GFP expression in M*eomesa*;M*hwa* double mutants (Figures 3C and D). The ratio of WT and single mutant embryos with strong GFP was decreased in the presence of SB. These results suggest that the *ndr1* regulatory regions used here harbor the necessary *cis*-elements required for responses to Eomesa, β-catenin, and Nodal autoregulation.

### Identification of regulatory regions of *ndr2 via* transgenesis

**FIGURE 4:**
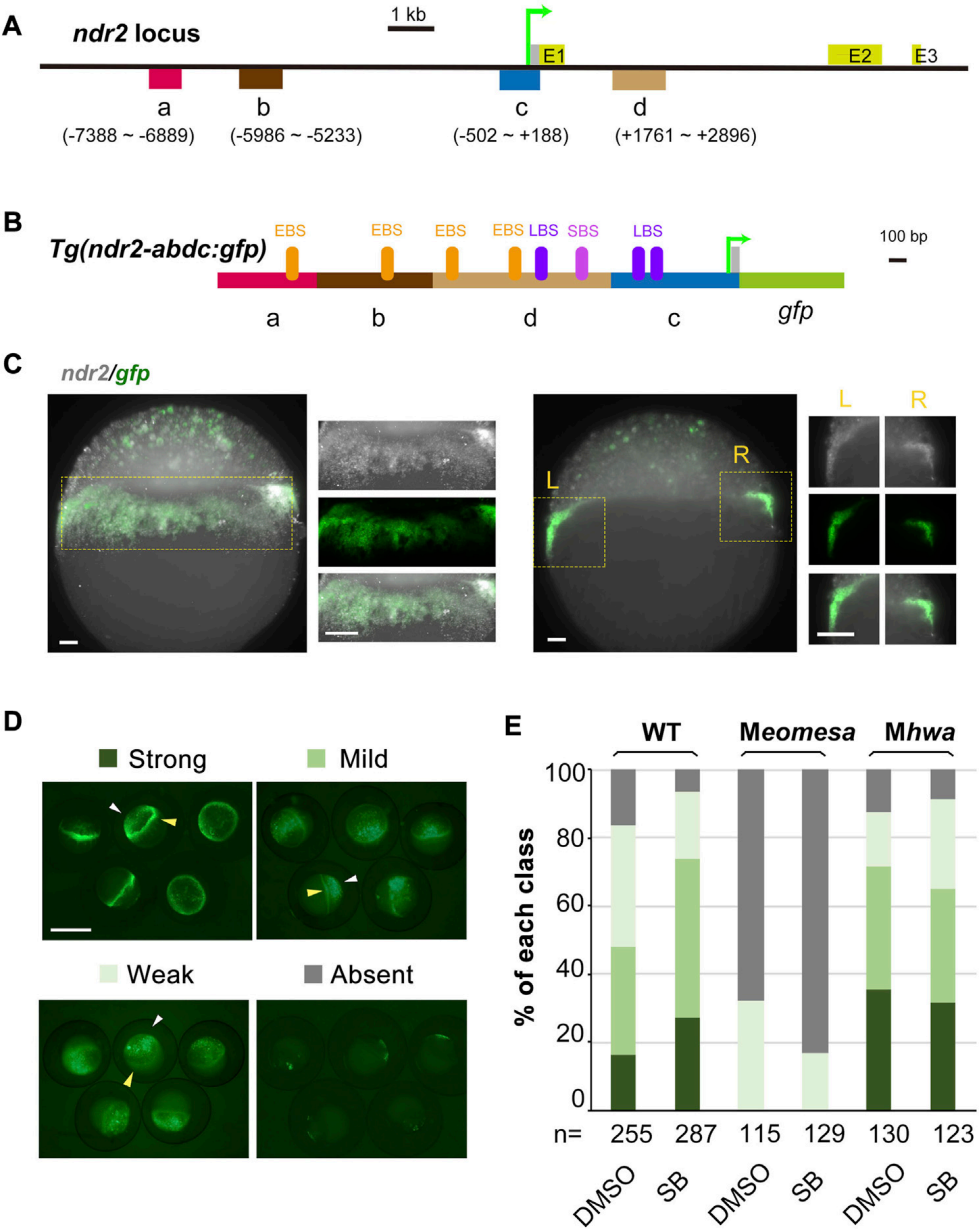
Verification of *ndr2 cis*-regulatory regions through transient transgenic assay. **(A–C)** Genomic organization of *ndr2* locus **(A)** and composition of transgenes **(B)** with specific *gfp* expression mimicking endogenous *ndr2* **(C)**. The translation start site was designated as position +1. Exons (E) of *ndr2* were indicated. EBS, Eomes-binding site; LBS, Lef/β-catenin binding site; SBS, Smad2-binding site. Wild-type embryos were injected with 50 pg *Tg(ndr2-abdc:gfp)* and harvested at about 30% epiboly stage for detection of *ndr2* and *gfp* expression pattern by FISH. Lateral views (the left panel) and vertical sections (the right panel) with magnification (indicated in the yellow rectangle) were shown. Scale bars: 50 μm. **(D–E)** Categorization of *ndr2* transgenes in zebrafish embryos. *Tg(ndr2-abdc:gfp)* DNA was injected into one-cell stage WT or mutant embryos (50 pg/embryo), and GFP was observed at 4.3–5 hpf. The embryo treatment and data presentation were similar to those described in Figure 3C and 3D.

For the *ndr2* locus, we selected four regions (a–d), which contain Eomes-binding peaks (ndr2R3 in the “a” region, ndr2R4 and ndr2R5 in the “b” region, ndr2R10 in the “c” region, and ndr211 in the “d” region) or Smad2-binding peaks (ndr2R11 and ndr2R12 in the “d” region), for making constructs (Figures 4A and B). The basal construct *Tg(ndr2-c:gfp)* consisting of the “c” region (proximal promoter with 5′UTR) and the *gfp* coding region and exhibiting universal GFP expression in the ectoderm (data not shown) was used to construct *Tg(ndr2-dc:gfp)*, *Tg(ndr2-adc:gfp)*, *Tg(ndr2-bdc:gfp)*, and *Tg(ndr2-abdc:gfp)*. Initial test results identified that only *Tg(ndr2-abdc:gfp)* gave rise to GFP expression recapitulating endogenous *ndr2* expression in the blastodermal margin but with nonspecific GFP in the ectoderm (Figure 4C). Then, we compared *Tg(ndr2-abdc:gfp)* expression in different genetic backgrounds and in the presence of SB (Figures 4D and E). The ratio of embryos with strong or mild GFP expression in the blastodermal margin in M*hwa* was comparable to that in the WT background, which was unaffected by SB treatment. However, only a small fraction (32%) of *Tg(ndr2-abdc:gfp)*-injected M*eomesa* embryos had weak GFP expression in the blastodermal margin, and this ratio was slightly reduced (to 17%) in the presence of SB. When injected into M*eomesa;*M*hwa* double mutants, GFP was hardly detectable. These changes were very similar to those seen for endogenous *ndr2* expression. Therefore, the a–d regions of *ndr2* harbor necessary *cis*-regulatory elements driving *ndr2* expression in the blastodermal margin. It is worth noting that *ndr2* regulatory elements for silencing *ndr2* expression in the ectoderm appear missing in the tested regions.

## Discussion

In this study, we applied causal graphical models to quantitatively explore the contribution of maternal Eomesa, maternal Hwa-activated β-catenin signaling, and Nodal autoregulation (Nodal/Smad2) to the dynamic expression levels of *ndr1* and *ndr2*, which revealed that Hwa/β-catenin and Nodal/Smad2 are the cause of *ndr1* expression, while maternal Eomesa and Nodal/Smad2 are the cause of *ndr2* expression. These causal graphical models were further explained by the *cis*-regulatory elements screening and verification in different genetic background mutants.

Gene transcription in eukaryotes requires the recruitment of RNA polymerase to promoter DNA through general transcription factors that assemble into a preinitiation complex (Zarrabi et al., 2018). Transcription factors are essential for promoter recognition and preinitiation complex formation and progression through the transcription cycle (initiation, elongation, and termination). For *ndr1* promoter recognition, two LBS and two EBS are predicted and located close together (Figure 3B), suggesting a potential interaction between β-catenin and Eomesa for reinforcing *ndr1* transcription. However, in case of a lack of either maternal Eomesa or Hwa/β-catenin, the *ndr1* transcripts could still be detected in the dorsal margin or whole margin (Figures 1A and B), suggesting the multiple toughening and protective mechanisms for *ndr1* transcription.

Although the *nodal* gene is believed to be activated by β-catenin in mice (Granier et al., 2011), Xenopus (Rex et al., 2002; Xanthos et al., 2002), and zebrafish (Kelly et al., 2000; Bellipanni et al., 2006), no direct evidence was presented. In zebrafish early embryos, β-catenin could directly bind to the promoter sequences of *ndr1*, not *ndr2* (Figures 2C and D), insinuating that the regulation of *ndr2* by β-catenin reported before may be mediated by Nodal autoregulation. There is a predicted LBS within the distal enhancer “a element” of *ndr1* identified before (Fan et al., 2007), but no binding activity of β-catenin in the range of “a element” (Figure 2C), and the deletion of “a element” of *Tg(ndr1-aIn1p:gfp)* did not change the expression pattern and levels of *gfp* (Figure 3B). Thus, the activation of *ndr1* by β-catenin may mainly depend on the LBS motifs located on the *ndr1* promoter (Figure 2C and Figures 3A and B).

The study of the *ndr2-gfp* transgenes provides new insights into the activation of *ndr2*. Previous studies found that the zygotic gene *mxtx2* could directly activate *ndr2* by binding to the first intron (Hong et al., 2011), which was further proved to be a potential enhancer element also bound by maternal Eomesa (Xu et al., 2014), but no *ndr2* promoter or transgenes was reported before. Here, four *ndr2* elements bound by Eomesa were screened out *via* ChIP-qPCR (Figure 3B) and further verified by transgenic constructs driving *gfp* expression, which could partially mimic endogenous *ndr2* expression, especially in the margin region (Figures 4A–C). The *ndr2-gfp* transgenes are hardly expressed under the loss of maternal Eomesa (Figures 4D–E), showing the indispensable role of maternal Eomesa for *ndr2* initiation.

In summary, our study explored the key cause of *ndr1* and *ndr2* activation among maternal Hwa/β-catenin signaling, maternal Eomesa, and Nodal autoregulation, and uncovered the underlying molecular mechanisms, which may help to understand the complicated and precise regulation of the *nodal* genes.

## Data Availability

The original contributions presented in the study are included in the article/Supplementary Material. All data and code about linear regression and causal inference are available for download at https://github.com/Starlitnightly/Analysis_Nodal. Further inquiries can be directed to the corresponding author.
