# Supplementary material for: Regulatory factor identification for nodal genes in zebrafish by causal inference
Source: Front Cell Dev Biol. 2022 Oct 20;10:1047363. doi: 10.3389/fcell.2022.1047363 (PMC9630340; doi:10.3389/fcell.2022.1047363)
Supplement: Supplementary file 1 [file Table1.pdf]

Supplementary tables

Table S1. The regression coefficient of the *ndr1* linear regression model

	coef	Std err	t	P>|t|	[0.025	0.975]
Intercept	8799.7445	3222.356	2.731	0.010	2227.707	1.54e+04
Time	-1036.6676	620.593	-1.670	0.105	-2302.375	229.040
maternal Eomesa	174.1414	1142.352	0.152	0.880	-2155.701	2503.983
Hwa/β-catenin	1627.4695	1142.352	1.425	0.164	-702.372	3957.312
Nodal/Smad2	4258.6067	932.726	4.566	0.000	2356.299	6160.915

Table S2 The regression coefficient of the *ndr2* linear regression model

	coef	Std err	t	P>|t|	[0.025	0.975]
Intercept	-7541.2095	4122.549	-1.829	0.077	-1.59e+04	866.785
Time	1934.2782	793.961	2.436	0.021	314.984	3553.572
maternal Eomesa	5501.2418	1461.478	3.764	0.001	2520.538	8481.946
Hwa/β-catenin	1131.3476	1461.478	0.774	0.445	-1849.356	4112.052
Nodal/Smad2	2531.4065	1193.292	2.121	0.042	97.672	4965.141

Table S3 Refutation test with adding a random common cause variable, replacing treatment with a random (placebo) variable, and removing a random subset of the data

	estimate	random_common_cause	placebo_treatment_refuter	data_subset_refuter
ndr1	2123.48	2144.51	0	2090.06
ndr2	7259.74	7211.40	0	7214.51

Table S4. Primers of *ndr1* for ChIP-qPCR

Number	Name	Sequence (5’ to 3’)
1	ndr1-9900-F	CAGCTGGGAGGGCATCTG
	ndr1-9900-R	CACCACAGCGGAATGAACTG
2	ndr1-9000-F	TGTGGAGGAAACTCGCATGA
	ndr1-9000-R	TCCTGGCTGGGTCAATTTCT
3	ndr1-8100-F	GCAGTGAGGTCAGGGCTTTATT
	ndr1-8100-R	AGGTAACGATCACCTGCATTTTC
4	ndr1-7000-F	TGTGTGTCGGTCAAGAATGGA
	ndr1-7000-R	GCGTGGTCTATTTTAGTTCCTCAAA
5	ndr1-6100-F	GCGCTGCAAATGGTTTGATT
	ndr1-6100-R	GGCAGACTTTGTAAGTGGAAAAACT
6	ndr1-5000-F	GGCGGGATCGATTTTGG
	ndr1-5000-R	GCATGGTGTCCACAACACTCTT
7	ndr1-3900-F	GCGATCTCTCCAGCTGCTGTA
	ndr1-3900-R	CCAGTGAGATTGTGCTGTTTGATT
8	ndr1-2900-F	TGGCCAAAACCAAAGGTGTT
	ndr1-2900-R	TCACAACTTGTCATGTATTTGAGCAT
9	ndr1-2000-F	CCCATGGGCTCATTGTAACC
	ndr1-2000-R	CCAACTTTATCCATCCTTCTCTCTTC
10	ndr1-1000-F	TGTTAAGAATGGCCAATGATGAA
	ndr1-1000-R	TTGTGGATTAAACACTGATCTGTCTGT

11	ndr1-180-F	TTTTCACACCTCCTGACGCA
	ndr1-180-R	ATACATGTTGGTGGGCCGTC
12	ndr1+900-F	GAAGCCCCTGCGAAGAAGT
	ndr1+900-R	AGGTTGAGTTAATCGGGTTTGC
13	ndr1+1900-F	AATGCGGCTGCCACTGA
	ndr1+1900-R	CGAGGAGCATATCCAAAGTGCTA
14	ndr1+2600-F	TCGATGTGCTCAGGATGCA
	ndr1+2600-R	TCTCGACATCAGCTAACACTGTTG

Table S5. Primers of *ndr2* for ChIP-qPCR

Number	Name	Sequence (5' to 3')
1	ndr2-10000-F	AACGTTTCAGCATCCATATTAC
	ndr2-10000-R	AATTCAGACTGGACTTTGCATATCTTT
2	ndr2-8700-F	CAGGGCAAAGCATAAAACAAATATT
	ndr2-8700-R	GCACAGCTATGTAAACTATTAAGATATACTTGATG
3	ndr2-7300-F	TCGTCGGGATCAATCGGTTC
	ndr2-7300-R	AACTCTTCTGGTGTTGGGGC
4	ndr2-5900-F	CCAATGGTTTAAAACTTGCCCTAAC
	ndr2-5900-R	TTTCTGAGTGCACTGATGTCAAAT
5	ndr2-5000-F	CCTCTGGGGCGGCAGAAT
	ndr2-5000-R	CGCCGGCCTCAACCCTAAAT
6	ndr2-4100-F	CCCCTGCTGGCCTCACTA

	ndr2-4100-R	CCTGGATGGGAGACCAAATG
7	ndr2-3000-F	CCCAGATTAAGTCGAAGGAAAGTTA
	ndr2-3000-R	TCCTTTAAACATTGCTGCTTTTGT
8	ndr2-2000-F	CAATTTATGAAAGCACATGCCAATA
	ndr2-2000-R	CACGTGTGCTGTCAAATTGATG
9	ndr2-1000-F	CCACCAACTTATCCAGCATATGTT
	ndr2-1000-R	GGACAATTTTAGCTTACCCGATTC
10	ndr2+1-F	AATGTCCTCTGCTACTTGTTAAATATGC
	ndr2+1-R	GAGTCCCGGGCGCTTTATA
11	ndr2+2000-F	TTTCGAGCCCGTGTTTACTC
	ndr2+2000-R	CGCTAACAAATCTCTCCGC
12	ndr2+3000-F	AAAGCGAGAAAGAGAGAGTG
	ndr2+3000-R	GATCATAAACCTATTTTATTGC
13	ndr2+4000-F	GGCTGACTGAAATTAAAA
	ndr2+4000-R	TTGCCTCTTCTTGTTGAATCATTA
14	ndr2+7000-F	CGGTTTTGTCGGAACGTCAG
	ndr2+7000-R	GAGCTCCAATGTCAGCCCAT
15	ndr2+8000-F	CAGAGAACACCACTGCACAAGAG
	ndr2+8000-R	CCATCCGATCTGGTTAAAATCC

Table S6. Primers for cloning *ndr1* and *ndr2* promoters

Primers	Sequence (5’ to 3’)
ndr1-i-F	agatctcgagctcaagcttGTAAGTATAATTGATTTTTTTAC
ndr1-i-R	tacttgtgttttcagacAGTGTTTAGGGCAGACAGG
ndr1-p-F	gtctgccctaaacactGTCTGAAAACACAAGTATAATTG
ndr1-p-R	gatccccgactgcagaattcGTCAAATCAAGGTAATAATC
ndr2-a-F	tagatctcgagctcaagcttACTCACAGACTAATGACGGCG
ndr2-a-R	ctctctcttgctcctcgaTTACACTCGCATCAACGACG
ndr2-b-F	cgtcgttgatgcgagtgtaaTCGAGGAGCAAGAGAGAGAG
ndr2-b-R	CGTCTTTTGTATGAGGCGTT
ndr2-c-F	CTGGCCTGTGTGAATTACCTT
ndr2-c-R	gatccccgactgcagaattcGGTATGCGCGTCTGGTTCAT
ndr2-d-F	aacgcctcatacaaaagacgTTTCGAGCCCGTGTTTACTC
ndr2-d-R	aaggtaattcacacaggccagCACTCTCTCTTTCTCGCTTT

Note that DNA bases in lowercase were chosen for overlapping PCR.
